# Flagellin-Deficient *Legionella* Mutants Evade Caspase-1- and *Naip5-*Mediated Macrophage Immunity

**DOI:** 10.1371/journal.ppat.0020018

**Published:** 2006-03-17

**Authors:** Tao Ren, Dario S Zamboni, Craig R Roy, William F Dietrich, Russell E Vance

**Affiliations:** 1 Genetics Department, Harvard Medical School, Boston, Massachusetts, United States of America; 2 Section of Microbial Pathogenesis, Yale University School of Medicine, New Haven, Connecticut, United States of America; Tufts University School of Medicine, United States of America

## Abstract

Macrophages from C57BL/6J (B6) mice restrict growth of the intracellular bacterial pathogen *Legionella pneumophila.* Restriction of bacterial growth requires caspase-1 and the leucine-rich repeat-containing protein Naip5 (Birc1e)*.* We identified mutants of L. pneumophila that evade macrophage innate immunity. All mutants were deficient in expression of flagellin, the primary flagellar subunit, and failed to induce caspase-1-mediated macrophage death. Interestingly, a previously isolated flagellar mutant *(fliI)* that expresses, but does not assemble, flagellin did not replicate in macrophages, and induced macrophage death. Thus, flagellin itself, not flagella or motility, is required to initiate macrophage innate immunity. Immunity to *Legionella* did not require MyD88, an essential adaptor for toll-like receptor 5 (TLR5) signaling. Moreover, flagellin of *Legionella* and *Salmonella* induced cytotoxicity when delivered to the macrophage cytosol using Escherichia coli as a heterologous host. It thus appears that macrophages sense cytosolic flagellin via a TLR5-independent pathway that leads to rapid caspase-1-dependent cell death and provides defense against intracellular bacterial pathogens.

## Synopsis

Legionella pneumophila is a bacterial pathogen that is the cause of a severe form of pneumonia known as Legionnaires' disease. A crucial aspect of the propensity of *Legionella* to cause disease lies in its ability to survive and multiply inside host immune cells known as macrophages. The intracellular survival and replication of *Legionella* can be studied using isolated macrophages grown in culture. Macrophages isolated from different laboratory mouse strains are differentially permissive for intracellular *Legionella* growth. This difference in permissiveness is genetic, and is conferred by differences in a mouse protein known as Naip5. The authors determined that *Legionella* strains that are unable to produce a protein called flagellin are able to grow inside normally resistant mouse macrophages. In addition, these flagellin^−^ strains are defective in initiating a cell-death response on the part of infected macrophages. Based on these data, the authors suggest that there is an intracellular mechanism for detecting the presence of bacterial flagellin protein, and that a cell-death response is initiated upon the detection of flagellin.

## Introduction

Legionella pneumophila is a motile gram-negative bacterium that is the cause of a severe form of pneumonia called Legionnaires' disease [1]. Virulence of *Legionella* depends on its capacity to replicate inside host macrophages. *Legionella* utilizes a type IV secretion system (encoded by the *dot/icm* genes) to inject effector proteins into the cytosol of host cells [2,3]. The secreted effector proteins (more than 30 of which have now been identified [4,5]) direct the host cell to create a unique intracellular vacuole in which *Legionella* can thrive and multiply [6,7].

Early studies found that macrophages from A/J mice support more than three logs of *Legionella* growth [8], whereas C57BL/6J (B6) macrophages are almost entirely restrictive. Remarkably, this large phenotypic difference is controlled by a single gene on mouse Chromosome 13 called *Naip5* (Neuronal apoptosis inhibitory protein 5, also called *Birc1e* [baculoviral IAP repeat-containing 1e]) [9,10]. Transgenic complementation experiments indicate that B6 mice encode a functional *Naip5* allele that confers resistance to *Legionella* [9,10]. A/J mice also express a *Naip5* gene; however, the A/J allele appears to be expressed at lower levels and encodes 14 amino acid polymorphisms as compared to the B6 allele. The precise polymorphism(s) within *Naip5* that is responsible for differential permissiveness to *Legionella* has yet to be identified.

Naip5 is a member of a superfamily of proteins that contain C-terminal leucine rich repeats (LRR) and a central nucleotide-binding domain (NBD) [11,12]. Evidence exists that many “NBD–LRR” proteins can function as microbe-detector proteins. For example, Nod1 and Nod2 confer recognition of bacterial cell wall–derived molecules [13–16] and Nod2 is required for resistance to orally delivered Listeria monocytogenes [17]. Human NALP1 is also reported to recognize muramyl dipeptide [18], and mouse *Nalp1* is required for susceptibility to anthrax lethal toxin [19]. *Ipaf,* the closest homolog of *Naip,* has been shown to be required for caspase-1 activation in response to *Salmonella* [20]. Although many NBD–LRR proteins appear to function in pathogen detection, there is no evidence that they bind directly to microbial products.

We hypothesized that B6 macrophages might restrict *Legionella* growth upon cytosolic detection of a *Legionella*-derived molecule. Here we identify flagellin in a genetic selection to identify essential elicitors of macrophage innate immunity. Our results suggest that flagellin initiates a caspase-1-dependent cell-death pathway that functions to restrict bacterial growth.

## Results/Discussion

### *Legionella* Flagellin Mutants Evade Macrophage Immunity

We adopted a genetic approach to identify *Legionella* gene products that might be recognized by host macrophages. We reasoned that *Legionella* strains lacking such a gene product might be able to evade macrophage immunity and replicate in normally restrictive B6 macrophages. A *mariner* transposon, which inserts with low bias into frequently occurring TA dinucleotides, was used to generate 30 independent pools of L. pneumophila transposon mutants. Each pool, containing approximately 2,500 mutants, was used to infect an independent well of B6 macrophages. After 6 d, we observed numerous bacteria in all the wells infected with transposon mutants, whereas virtually no bacteria were observed in wells infected with unmutagenized *Legionella.* Sixty-four transposon mutants obtained from one round of selection in B6 macrophages were retested and, of these, 60 mutants (93.8%) were found to be capable of robust growth in B6 macrophages. Thus, growth in B6 macrophages represents a powerful and highly efficient selection procedure.

The gene disrupted by the transposon was determined for 29 independent mutants. Twenty-four mutants were found to have insertions within the *flaA* open reading frame, four mutants were found to harbor insertions within the *flaA* promoter, and one mutant (with a weaker growth phenotype, unpublished data) was recovered with an insertion in *fliA,* a transcriptional activator of *flaA* [21]. The *flaA* gene encodes flagellin, the main structural subunit of flagella. Our screen was highly saturating, with an average of approximately 25 transposon insertions per *Legionella* gene, representing an average of one insertion per 45 bp. We conclude that *flaA,* and not genes encoding other components of the flagellar regulon, is the primary locus into which a transposon insertion can permit evasion of innate macrophage defenses that normally restrict bacterial growth.

**Figure 1:**
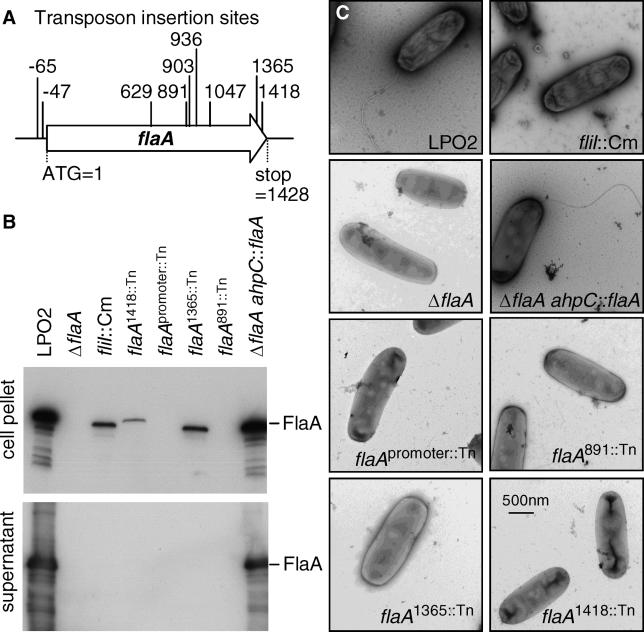
Characterization of *Legionella* Mutants Harboring Transposon Insertions in Flagellin *(flaA)* (A) Nucleotide positions of transposon insertions in *flaA.* (B) Western blotting for flagellin (FlaA). Proteins from pelleted bacteria (top) or TCA-precipitated culture supernatants (bottom) obtained from stationary-phase cultures were separated by SDS-PAGE, blotted, and probed with an anti-FlaA monoclonal antibody. Δ*flaA ahpC::flaA* is the Δ*flaA* mutant which has been complemented with a copy of *flaA* inserted on the *Legionella* chromosome just after the *ahpC* locus. (C) Electron microscopy showing the presence or absence of flagella on various *Legionella* strains. Representative bacteria (of >100 surveyed) are shown. No flagellated bacteria were seen for any of the strains shown as lacking flagella. Tn, transposon.

The precise transposon insertion site was determined for nine mutants (Figure 1A), of which four were selected for further analysis. LP02 *flaA* ^promoter::Tn^ harbors a transposon insertion 47 bp upstream of the *flaA* start codon. LP02 *flaA* ^891::Tn^, *flaA* ^1365::Tn^, and *flaA* ^1418::Tn^ harbor insertions at nucleotides 891, 1,365, and 1,418 of the open reading frame, respectively. The latter strain is of particular interest since the transposon inserted only 10 bp from the stop codon. Translation read-through into the transposon is predicted to result in the last two amino acids of FlaA being replaced with a 37 amino-acid peptide.

### All Mutants Evading Macrophage Defenses Are Nonflagellated and Nonmotile

In order to confirm the results of the transposon mutagenesis, an unmarked deletion of *flaA* was generated and then complemented by a chromosomal copy of *flaA.* For comparison, we also obtained a previously characterized *fliI* mutant [22]. *FliI* encodes an ATPase required for flagellar secretion and assembly. The *flaA* ^promoter::Tn^, *flaA* ^891::Tn^, and the Δ*flaA* strains did not detectably express FlaA protein (Figure 1B), whereas the *fliI, flaA* ^1365::Tn^, and *flaA* ^1418::Tn^ strains expressed *flaA* but were defective in the secretion of FlaA to the culture supernatant (Figure 1B). Owing to its extended open reading frame, the FlaA protein expressed by LP02 *flaA* ^1418::Tn^ exhibited a higher molecular weight (Figure 1B). As expected, LP02 and the Δ*flaA ahpC*::*flaA* (complemented) strains were motile (unpublished data). The four transposon mutants examined were all nonmotile, as were the unmarked Δ*flaA* mutant and the *fliI* mutant (unpublished data). Each mutant was also examined by electron microscopy (Figure 1C). LP02 and the Δ*flaA ahpC*::*flaA* (complemented) strains were found to exhibit normal flagella, whereas the *fliI* mutant and all the *flaA* mutants were completely nonflagellated.

### Flagellin Itself Is Required for Restriction of Bacterial Growth

We tested the ability of various mutants to grow in bone marrow–derived macrophages. In addition to using B6 macrophages, which express a fully functional allele of *Naip5* and therefore normally restrict *Legionella* growth, we also used a consomic mouse strain, B6.A-Chr13, which is B6 at all loci (~95%), except for those that lie on Chromosome 13, which is derived from the A/J strain of mouse. Since Chromosome 13 harbors the *Naip5* locus, both A/J and B6.A-Chr13 mice carry the A/J allele of *Naip5* and are permissive for *Legionella* growth.

**Figure 2:**
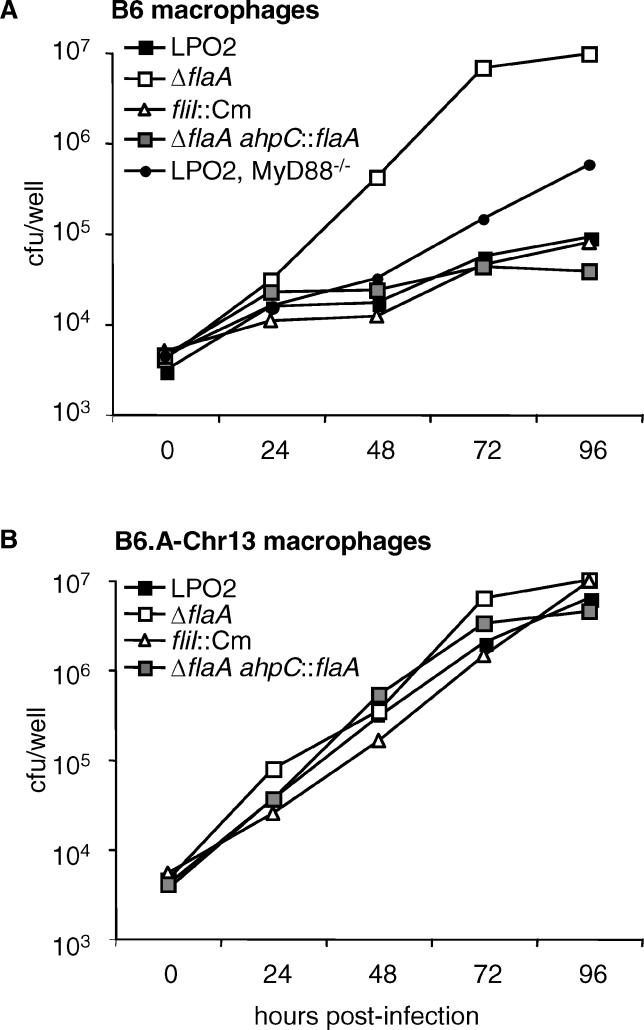
Growth of *Legionella* in Bone Marrow–Derived Macrophages Growth of *Legionella* strains in bone marrow–derived macrophages from (A) B6 mice or (B) B6.A-Chr13 mice (which carry the permissive A/J allele of *Naip5*). A confluent layer of macrophages were grown in 24-well plates and were infected with the indicated *Legionella* strains at an MOI of 0.02. Growth of wild-type *Legionella* (LP02) in B6-backcrossed *MyD88^−/−^* macrophages is also depicted in (A). After addition of bacteria, the plate was spun at 400 *g* for 10 min. One hour after infection, the media was changed. At daily intervals (starting with 1 h post-infection), a timepoint was taken. Macrophages were lysed with sterile water and the lysate combined with supernatant from the same well. Colony-forming units per well were determined by plating dilutions onto BCYE plates.

As expected, the Δ*flaA* strain recapitulated the phenotype of the transposon mutants and grew robustly in B6 macrophages, indicating that it is indeed loss of *flaA,* and not polar or unlinked mutations, which results in the growth phenotype (Figure 2). This observation confirms identical results obtained independently by the Swanson group at the University of Michigan (Ari Molofsky and Michele Swanson, personal communication). In contrast, the Δ*flaA ahpC*::*flaA* (complemented) strain was restricted in growth in B6 macrophages, but grew well in permissive B6.A-Chr13 macrophages. The *fliI* mutant (which expresses but does not secrete or assemble flagellin) failed to grow in B6 macrophages (Figure 2A), but grew in B6.A-Chr13 macrophages (Figure 2B), suggesting that innate immunity to *Legionella* is triggered by FlaA itself, and not by motility or flagella. Interestingly, although the *fliI, flaA* ^1365::Tn^, and *flaA* ^1418::Tn^ strains expressed similar intracellular levels of FlaA protein (Figure 1), only the *fliI* mutant was growth-restricted. We conclude that transposon insertions at the very C-terminus of *flaA* must affect secretion of flagellin into host cells or subsequent flagellin recognition by host cells.

### Macrophage Immunity Is Independent of Toll-Like Receptor 5

Flagellin stimulates immune signaling through toll-like receptor 5 (TLR5) [23], and there is evidence that TLR5 plays an important role in controlling *Legionella* infections in vivo [24]. However, mouse macrophages do not express TLR5 [25]. Thus, it is unlikely that TLR5 plays a role in mouse-macrophage restriction of *Legionella* growth in our in vitro system. This is difficult to prove directly since TLR5 knockouts have not yet been reported. Nevertheless, we sought to confirm that macrophage recognition of flagellin does not require TLR5 by examining the phenotype of macrophages deficient in MyD88, a signaling adaptor required for all TLR5 signaling [23]. After 48 h of growth, B6 *MyD88^−/−^* macrophages restricted *Legionella* growth as well as wild-type macrophages (Figure 2A), implying that TLR5 is not required for the initial restriction of flagellin^+^ bacteria. Interestingly, by 96 h, *MyD88^−/−^* macrophages exhibited more growth of wild-type *Legionella* than did B6 macrophages, and this is likely a consequence of reduced MyD88-dependent signaling (e.g., NF-κB activation), downstream of other toll-like receptors that may play a role in the later phases of infections.

### Flagellin Mediates Caspase-1-Dependent Cytotoxicity of B6 Macrophages

The mechanism by which flagellin-mediated signaling restricts *Legionella* growth in B6 macrophages is unknown. However, a recent study demonstrated that wild-type *Legionella* activates caspase-1, and that caspase-1 is required for restriction of *Legionella* growth [26]. Caspase-1 does not mediate classical apoptosis but is instead required for processing of pro-IL-1 and pro-IL-18 into their mature (secreted) forms [27,28]. Since we found that addition of exogenous IL-1 and/or IL-18 did not render macrophages resistant to *Legionella* (unpublished data), we concluded that caspase-1 is required for host defense via a mechanism that is independent of IL-1 and/or IL-18. Interestingly, in response to several stimuli, including *Salmonella* [29,30], *Shigella* [31], and anthrax lethal toxin [19], caspase-1 has also been shown to be required for an unusual form of macrophage cell death that is distinguishable from apoptosis by its extremely rapid time-course (<4 h) and its inflammatory/necrosis-like characteristics [32]. We therefore considered the possibility that flagellin promoted host defense by activating rapid host cell death.

**Figure 3:**
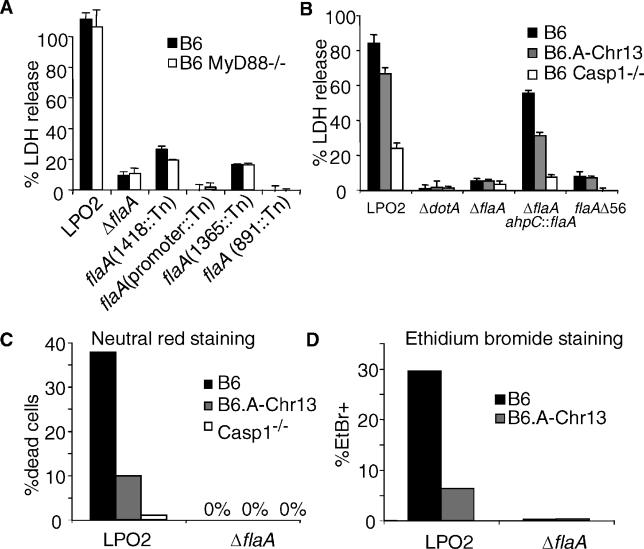
Rapid Lysis of Macrophages in Response to Flagellin-Expressing *Legionella* In all experiments shown, bacteria were added at an MOI of 2, and were spun onto the macrophages at 400 *g* for 10 min. Cells were assayed 4 h after infection. LP02 is the wild-type strain and is isogenic with the mutants utilized in these experiments. (A) Release of the intracellular enzyme LDH by B6 and *MyD88^−/−^* macrophages with indicated *Legionella* strains, including four mutants with transposon insertions in *flaA*. One hundred percent release is set as the amount of LDH released by detergent-treated macrophages (minus spontaneous release). (B) LDH release by B6, B6.A-Chr13, and B6 caspase-1-deficient macrophages infected with the indicated *Legionella* strains. FlaAΔ56 is an LP02-derived strain that contains an EMS-induced point mutation in *flaA,* resulting in a truncation of the last 56 amino acids of FlaA. (C) Cell death of macrophages was assessed by degree of failure to take up neutral red in a 4-h assay. At least 100 macrophages were counted for each condition; one representative experiment of two is shown. (D) Cell death was quantified by assessing permeability to ethidium bromide-2 homodimer. At least 400 macrophages were counted for each condition; one representative experiment of two is shown.

Macrophages dying due to caspase-1 activation rapidly lose plasma membrane integrity and can be detected by their release of the intracellular enzyme lactate dehydrogenase (LDH), by their failure to take up neutral red by micropinocytosis, or by their increased permeability to ethidium bromide. Initially, we tested the Δ*flaA* mutant and our transposon mutants for the ability to initiate macrophage death of B6 macrophages in a 4-h LDH-release assay. Remarkably, we observed that the Δ*flaA* mutant and the strains with transposon insertions in *flaA* were all noncytotoxic, as compared to the wild-type LP02 strain (Figure 3A). *MyD88^−/−^* macrophages behaved similarly to wild-type macrophages (Figure 3A), implying that flagellin-induced cell death, like restriction of bacterial growth, does not depend on TLR5. In contrast, caspase-1-deficient macrophages, which are permissive for *Legionella* growth [26], exhibited marked resistance to *Legionella*-induced death (Figure 3B and 3C). The Δ*flaA* mutant was confirmed to be noncytotoxic by two other assays of rapid cell death: failure to take up neutral red (Figure 3C) and inability to exclude ethidium bromide (Figure 3D). Our results reveal a striking correlation between the induction of cytotoxicity (seen only with *flaA* ^+^ bacteria infecting wild-type or *MyD88*-deficient macrophages) and restriction of bacterial replication.

### *Naip5* Genotype Is Associated with Resistance to Flagellin-Induced Death

**Figure 4:**
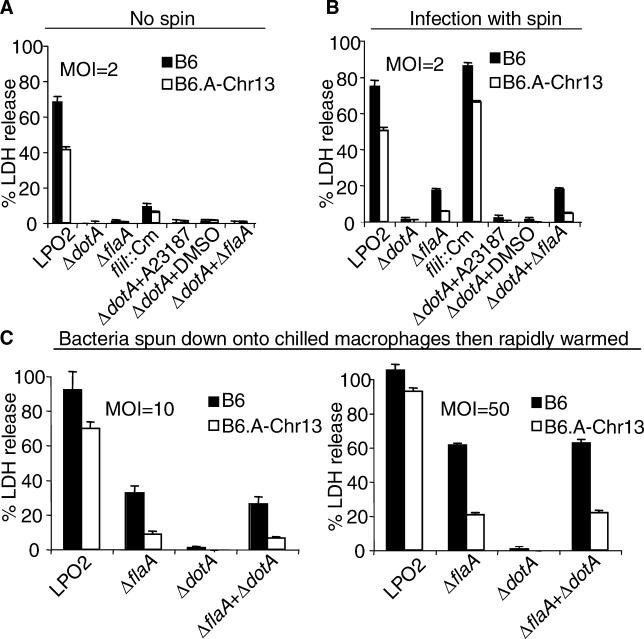
Flagellin-Induced Cytotoxicity of Macrophages Is Independent of Motility but Requires Type IV Secretion (A and B) Centrifugation is required to promote cytotoxicity of a *fliI* mutant, but does not restore cytotoxicity of a *flaA* mutant. The calcium ionophore A23187 (1 μM, or a vehicle control, DMSO) was added to some wells simultaneously with the addition of bacteria. Infection and LDH release were assayed as in Figure 3, with or without centrifugation immediately after infection, as indicated. (C) Coinfection of macrophages with a Δ*flaA* and Δ*dotA* mutant does not restore cytotoxicity. Infection and LDH release were assayed as in Figure 3, except that higher MOIs were used and chilled macrophages were infected, as indicated.

If rapid macrophage death is required for resistance to *Legionella* growth, a simple prediction is that macrophages permissive for *Legionella* growth (e.g., from A/J or B6.A-Chr13 mice) should be resistant to flagellin-mediated cytotoxicity. As predicted, flagellin^+^ bacteria killed a larger percentage of B6 macrophages (as assessed by failure to take up neutral red; Figure 3C), as compared to macrophages carrying the A/J allele of *Naip5*. A significant difference between B6 and B6.A-Chr13 macrophages in susceptibility to *Legionella*-induced cell death was also observed when cell death was measured by permeability to ethidium bromide (Figure 3D). B6 macrophages also showed greater release of LDH as compared to susceptible B6.A-Chr13 macrophages (Figures 3B and 4), though the difference was not always dramatic, possibly because the LDH-release assay is not a single-cell assay and does not distinguish between generalized nonlethal leaking of cells and individual cell death. Thus, although B6.A-Chr13 mice were significantly resistant to flagellin-mediated killing, resistance was not complete, suggesting that the A/J-derived *Naip5* allele in these mice is likely not a complete null. Consistent with these results, Molofsky et al. [33] also observed significant *flaA*-dependent killing of A/J macrophages.

### Motility Is Not Required to Induce Cytotoxicity

In the cytotoxicity experiments, we usually promoted contact between *Legionella* and macrophages by centrifugation (400 *g* for 10 min). This was done because we and others [33,34] have observed that nonmotile bacteria exhibit reduced cytotoxicity simply because they fail to contact macrophages in sufficient numbers. For example, the nonmotile but flagellin-positive *fliI* mutant appeared non-cytotoxic when infection was initiated without a spin (Figure 4A). Importantly, however, centrifugation restored complete cytotoxicity to the *fliI* mutant (Figure 4B), but had virtually no effect on the cytotoxicity of the Δ*flaA* mutant. Thus, consistent with the growth assays (Figure 2), flagellin itself, and not motility or flagella, appears to be key for initiating macrophage innate immunity.

### Flagellin-Induced Cytotoxicity Requires Type IV Secretion

It has been previously reported that bacteria harboring mutations in the type IV (dot/icm) secretion apparatus are noncytotoxic [7,35]. It has been proposed that type IV–dependent toxicity, usually seen at high multiplicities of infection (MOIs), is due to pores induced in the host membrane by the dot/icm secretion apparatus [36]. We found that even at relatively low MOI, flagellin-induced killing of macrophages was dot/icm–dependent (Figures 3B and 4B).

A trivial explanation of our data is that flagellin is required for expression or function of the dot/icm apparatus, but this model cannot be easily reconciled with the fact that the dot/icm apparatus is strictly required for intracellular growth and the observation that flagellin mutants exhibit robust intracellular growth. Thus we considered two other non-mutually exclusive models: (1) a “secretion model” in which flagellin is secreted into host cells via the type IV (dot/icm) apparatus, or leaks into the cytosol via pores in the phagosome generated by the dot/icm apparatus; or (2) a “two signal” model, in which puncture of the cell membrane by the dot/icm apparatus provides a signal that is independent of that provided by flagellin, and both signals are required to initiate cell death. In the two-signal model, the signal provided by the dot/icm apparatus might be the pores themselves, a pore-induced ion flux, or a pore-induced leakage of microbial products, such as peptidoglycan fragments [37], into the cytosol.

We tested the “two signal” model in several ways. First, to test whether the dot apparatus induces a calcium flux that (in combination with flagellin) induces cell death, we infected macrophages with *dotA* mutant (but flagellin^+^) bacteria in the presence of A23187, a calcium ionophore. A23187 did not restore cytotoxicity to the *dotA* mutant (Figure 4A and 4B) and, moreover, pretreatment of cells with thapsigargin (to deplete intracellular calcium stores [38]) failed to protect macrophages from cytotoxicity of wild-type bacteria (unpublished data). To test whether a dot/icm–dependent, flagellin-independent but non–calcium-mediated signal was required for cell death, we coinfected macrophages with *dotA* and *flaA* mutant bacteria. If the dot/icm apparatus and flagellin provide two independent signals required for cell death, we predicted that coinfection with both mutants should restore cytotoxicity, but this was not the case (Figure 4A and 4B). Even at high MOIs (Figure 4C), where each macrophage was infected with multiple bacteria of each genotype, there was no synergy between *flaA* and *dotA* mutants in induction of cell death. Thus, the “two signal” model is not sufficient to account for our observations, and it appears likely that one role of the dot/icm apparatus in induction of cytotoxicity is to permit flagellin access to the cytosol. It was interesting that at very high MOIs, the *flaA* mutant exhibited significant apparent toxicity. The basis for this toxicity is unclear, but it appears to depend on the dot apparatus and may represent a “pore-forming” cytotoxicity [36] that is revealed at high MOIs.

In the above coinfection experiments, the *dotA* and *flaA* mutants were not frequently present in the same phagosome (unpublished data), even using specialized infection procedures [39] (Figure 4C) and high MOIs. Therefore we could not distinguish whether flagellin is directly transported into the cytosol via the dot/icm secretion apparatus (in *cis*), or merely leaks into the cytosol via dot/icm–induced pores in the phagosome (in *trans*). Nevertheless, we favor the idea that the dot/icm apparatus translocates flagellin into the host cell cytosol, since we observed that *fliI* mutants retain cytotoxicity, despite the fact that *fliI* is required for flagellar secretion (Figure 1) [22,33]. Although another secretion system might be responsible for secretion of flagellin into the host cell cytosol, the type IV secretion system seems the most likely candidate.

### *Salmonella* Flagellin Also Induces Death of B6 Macrophages

**Figure 5:**
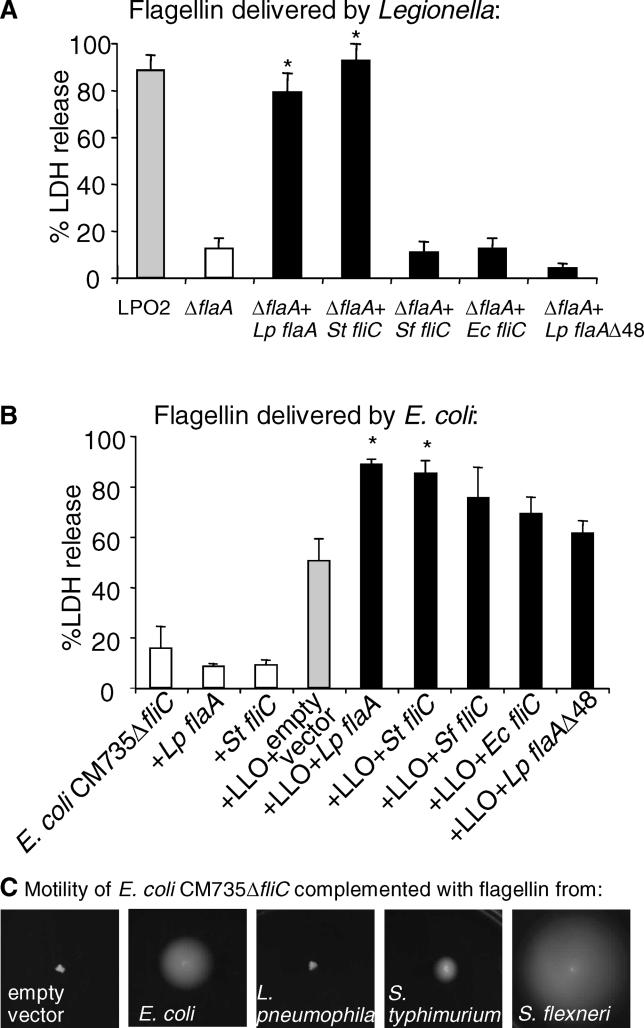
*Salmonella* and *Legionella* Flagellin Induce Macrophage Death (A) *Salmonella* flagellin complements a Δ*flaA* mutant of *Legionella.* The LP02 Δ*flaA* mutant was complemented with plasmids expressing flagellin of *L. pneumophila (Lp flaA), S. typhimurium (St fliC), S. flexneri (Sf fliC),* or *E. coli (Ec fliC).* A 48-amino acid C-terminal truncation mutant of FlaA (*Lp flaA*Δ48) was also expressed as a control. Infection and LDH release of B6 macrophages were assayed as in Figure 3. An MOI of 2 was used in this experiment. Asterisks denote *p* < 0.02 (two-tailed student's *t*-test) versus Δ*flaA* + *Lp flaA*Δ48*.* (B) Flagellin from *Legionella* and *Salmonella* induce macrophage death when delivered to the cytosol by *E. coli.* A flagellin-deficient strain of E. coli (CM735Δ*fliC*) was transformed with the same plasmids as in (A), or an empty vector as a control. In some cases, the E. coli also expressed a non-secreted form of the pore-forming toxin LLO. In this system, E. coli is degraded in the phagolysosome, releasing cytoplasmic LLO. LLO forms pores in the phagosome, giving phagosomal contents access to the cytosol. Infection and LDH release of B6 macrophages was assayed as in Figure 3. An MOI of 2 was used in this experiment. Asterisks denote *p* < 0.02 (two-tailed student's *t*-test) versus LLO + empty vector (gray bar). The *p*-value for LLO + Sf *fliC* was 0.05; the *p*-value for LLO + Ec *fliC* was 0.05; and the *p*-value for LLO + Lp FlaAΔ48 was 0.155. (C) The E. coli CM735Δ*fliC*-derived strains from (B) were tested for the ability to swim through soft agar (motility) plates. Nonmotile strains remain at the point of inoculation, whereas motile strains show a halo of bacteria swimming outward from the point of inoculation.

We complemented the *Legionella* Δ*flaA* mutant with flagellin *(fliC)* from *Salmonella, Shigella,* and *Escherichia coli,* as well as with flagellin *(flaA)* from *Legionella.* Remarkably, we found that *Salmonella* flagellin was able to complement the *Legionella* Δ*flaA* mutation for cytotoxicity of B6 macrophages (Figure 5A), despite only 59% similarity at the amino acid level. Interestingly, both *Salmonella* and *Legionella* flagellins contain two leucines near their C-termini, a potential signal for recognition by the *Legionella* type IV secretion system [40]. Complementation of the Δ*flaA* mutant with a *Legionella* FlaA protein lacking its final 48 amino acids failed to restore cytotoxicity, as did complementation with E. coli or *Shigella* flagellin (Figure 5A). We do not know why these other flagellins failed to complement cytotoxicity, but it is possible that the flagellin proteins from these species are not recognized, or that expression and/or delivery of these heterologous flagellin proteins are low. However, our findings suggest a partial molecular explanation for the ability of *Salmonella* to induce rapid, caspase-1-dependent death of macrophages (see below).

An E. coli strain (CM735) carrying an in-frame deletion of flagellin *(*Δ*fliC)* was used as a heterologous host to express *Legionella* and *Salmonella* flagellin. CM735 does not express a type III or type IV secretion system capable of delivering proteins to the host cytosol and, as expected, *Legionella* or *Salmonella* flagellin did not confer cytotoxicity to E. coli (Figure 5B). This result suggests that flagellin must be delivered to the cytosol in order to induce cytotoxicity. To test this, E. coli CM735Δ*fliC* was transformed with a plasmid encoding listeriolysin O (LLO), a cholesterol-dependent pore-forming toxin that *Listeria* utilizes to disrupt phagosomal (but not plasma) membrane [41]. Expression of LLO itself caused some toxicity of macrophages (Figure 5B), but this was markedly increased when the *E. coli* strain also expressed *Legionella* or *Salmonella* flagellin (*p* < 0.02; Figure 5B). Delivery of Shigella flexneri or E. coli flagellin induced weak but statistically insignificant cytotoxicity (*p* = 0.05), whereas a truncated form of *Legionella* flagellin (lacking the C-terminal 48 amino acids) was noncytotoxic. The results imply that *Legionella* (or *Salmonella*) flagellin does not require other *Legionella* (or *Salmonella*) proteins in order to induce cytotoxicity of macrophages. In addition, since the E. coli strain expressing *Legionella* flagellin was entirely nonmotile, as assessed by movement through soft agar (Figure 5C), the results reconfirmed that bacterial motility is not required for induction of macrophage death.

### Conclusions

Our data are consistent with a model in which rapid caspase-1-dependent macrophage death, induced in B6 macrophages by flagellin, leads to restriction of *Legionella* growth. Caspase-1 activation has been proposed to promote immune defense by initiating rapid release of inflammatory cytokines (IL-1 and IL-18). Although such inflammation may sometimes [42] (but not always [43]) provide immune defense in vivo, our results suggest that caspase-1-mediated death can also play a more direct role in restricting bacterial growth.

How might rapid cell death restrict *Legionella* growth? We propose that two effects may be in play. First, it is well established that after infection of a macrophage, *Legionella* delays replication for several hours until a proper replicative vacuole can be established [44]. Second, during the late phases of replication in susceptible cells, *Legionella* is known to upregulate gene products (e.g., the dot/icm apparatus) that help promote infectivity for new host cells [45]. Thus, a macrophage that undergoes rapid cell death will not only release fewer bacteria, but each released bacterium may also exhibit reduced infectivity for a new host cell. These two effects, in combination with other potential mechanisms [46], may lead to the significant growth restriction observed in B6 macrophages.

If our model is correct, and *Legionella* flagellin is sensed by host cells, an obvious question is: what host protein is responsible for the sensing? We have provided evidence that sensing of flagellin, rapid macrophage death, and growth restriction of *Legionella* appear to be independent of TLR5. We consequently favor the idea that a novel intracellular sensor protein may be responsible for sensing flagellin. An obvious candidate is Naip5, since this protein is known to be required for restriction of *Legionella* growth, and the *Naip5* genotype correlates with the ability to sense flagellin (Figure 3). However, a recent study [26] demonstrated that the *Naip5* homolog, *Ipaf,* is also required for restriction of *Legionella* growth, and moreover, *Ipaf* has been shown to be critical for activation of caspase-1 in response to *Salmonella* [20]*.* Caspase-1 is also regulated by the “inflammasome”, a complex of proteins including Nalp family members [47]. Higher-order complexes containing Naip, Nalp, Ipaf, and/or caspase-1 may exist and, thus, it is possible that Ipaf, Nalp, or another host protein is the sensor of flagellin, and that Naip5 plays an important role as a subsequent regulator of caspase-1 activation. For the future, analyzing a null allele of *Naip5* may provide more specific information about its role in the caspase-1-mediated cell-death pathway than studying the naturally occurring A/J variant.

We do not anticipate that it will be easy to prove direct recognition of flagellin by a host receptor. Indeed, extensive characterization of the much more intensively studied toll-like or Nod-like receptors has yet to provide uncontestable direct evidence of binding to a bacterial ligand. We believe that it is likely that other bacterial products, such as peptidoglycan, will also induce macrophage death when delivered to the cytosol. Therefore, conclusive evidence that flagellin itself is sensed, as opposed to a flagellin-associated contaminant, will be difficult to obtain until direct binding assays with purified proteins are developed, and a source of recombinant flagellin, expressed in the absence of bacteria, is utilized.

In addition, there is the distinct possibility that flagellin itself is not sensed, but that the presence of flagellin imparts some physiological change on host macrophages, and it is this physiological alteration that is sensed. It is also possible that flagellin imparts a physiological change to *Legionella* that renders it more cytotoxic to macrophages. This latter possibility seems unlikely given the ability of *Legionella* flagellin to induce cell death when delivered to the macrophage cytosol by *E. coli.*

Finally, irrespective of mechanism, we believe our findings may be relevant not only to *Legionella,* but also for understanding immune defense against a variety of flagellated pathogens. Along these lines, we found that *Salmonella* flagellin is also capable of inducing rapid macrophage cell death (Figure 5). It is well established that *Salmonella* induces caspase-1-dependent death of macrophages [48]. It has been proposed that the *Salmonella* type III–secreted effector SipB binds to caspase-1 [30], leading to caspase-1 activation, but this conclusion has been challenged for a variety of reasons [48]. In fact, *Salmonella*-induced macrophage death may be caused by several redundant mechanisms. Thus, it seems plausible that flagellin, perhaps delivered to the cytosol by the *Salmonella* type III secretion system, may be responsible, at least in part, for caspase-1-dependent toxicity induced by *Salmonella.*

Consistent with this model, we found that a *Salmonella* strain unable to express either of two flagellin genes *(fljB fliC)* had a reduced cytotoxicity similar to a type III *invA* secretion mutant (Figure S1A). This experiment must be interpreted with caution, however, because the invasiveness of the flagellin mutants is visibly less than that of the wild-type, higher MOIs tend to increase the cytotoxicity of the flagellin mutants (Figure S1B), and we did not perform experiments with a control strain equivalent to the *Legionella fliI* mutant (flagellin^+^, but flagellum^−^). However, we do note that one report demonstrated that mutations in *fliA* (required for flagellin expression) but not *flgK* (required for flagellar assembly) resulted in a motility-independent defect in induction of rapid macrophage cytotoxicity [34].

Interestingly, we did not observe differences between B6 and B6.A-Chr13 macrophages in their cytotoxicity response to *Salmonella* infection (Figure S1). This is not very surprising, since variations in mouse *Salmonella* susceptibility have been mapped in several instances, and have never been linked to the *Lgn1* locus. Based on this information, we conclude that the *Naip5* allelic variations found in B6 and A/J may not cause differential responses to intracellular flagellins from *all* flagellated pathogens.

Although many questions remain, our unbiased genetic mutagenesis and selection has identified *Legionella* flagellin (but not the flagellum) as a key determinant in initiating innate immune-defense mechanisms, including caspase-1-dependent macrophage cell death and restriction of bacterial growth. We believe our results provide insights for understanding macrophage resistance to intracellular bacterial pathogens.

## Materials and Methods

### Mice.

B6 and consomic B6.A-Chr13 mice were purchased from The Jackson Laboratory (Bar Harbor, Maine, United States). Two independently derived caspase-1-deficient mice lines [27,28] were used and gave similar results. *MyD88^−/−^* mice, backcrossed at least five times to B6, were obtained from the Medzhitov Laboratory (Yale University School of Medicine). All knockout mice were confirmed to be homozygous for the B6 *Naip5* locus.

### Bacterial strains.

LP02 is a streptomycin-resistant thymidine auxotroph derived from L. pneumophila LP01. An unmarked deletion of *flaA* (corresponding to nucleotides 1478105–1479574 of the LP01 genome [49]) was generated in LP02 by use of the allelic exchange vector pSR47S [22]. The Δ*flaA* strain was also complemented by inserting the *flaA* gene and its own promoter (corresponding to nucleotides 1478136–1479915 of the LP01 genome) on the chromosome just after the *ahpC* gene (at position 3354877 of the LP01 genome). The *ahpC* locus is highly expressed but is not essential for *Legionella* virulence [50]. The *fliI* null strain LP02 *fliI*::Cm [22] was the kind gift of R. Isberg (Tufts University School of Medicine). The broad-host-range plasmid pBBR1-MCS2 was used to express flagellin from *Legionella, E. coli* MG1655 (*fliC,* b1923), S. flexneri 2457T (*fliC,* S2062), and Salmonella typhimurium LT2 (*fliC,* STM1959). The various flagellin open reading frames were first cloned into pET28a (NcoI*,* XhoI) and then transferred to pBBR1-MCS2 (XbaI*,* PvuI), such that all flagellins were expressed from the *lac* promoter of pBBR and the ribosome-binding site of pET28a. Expression was induced with 1mM IPTG. The pBBR-flagellin constructs were also expressed in E. coli CM735Δ*fliC* [51], along with LLO (pACYC184-LLOc) [41]. *Salmonella* strains were on the LT2 background and were the kind gift of the Starnbach laboratory.

### Transposon mutagenesis.

A *mariner* transposon (pSC123, kind gift of S. Chiang and J. Mekalanos) was mated from E. coli strain SM10λpir into L. pneumophila strain LP02. Eight separate matings were plated on a total of 30 buffered charcoal yeast extract (BCYE) plates containing streptomycin (50 μg/ml) and kanamycin (25 μg/ml). Approximately 2,500 colonies from each independent plate were pooled and used to infect bone marrow–derived macrophages from B6 mice at an MOI of 0.05. Unmutagenized LP02 was used to infect macrophages as a control. After 6 d, bacteria were visible in each well that had been infected with mutagenized LP02, but virtually no bacteria were evident in wells that had been infected with unmutagenized LP02. Bacteria were harvested from each well, plated on BCYE plates, and individual colonies were tested for the ability to grow in B6 macrophages. The transposon insertion site for selected strains was determined by arbitrary PCR.

### Arbitrary PCR.

Crude DNA from a single *Legionella* colony was used in a first round of PCR with primers JZ320–1 (GCCCGGGAATCATTTGAAG), ARB1 (GGCCACGCGTCGACTAGTACNNNNNNNNNNGATAT), and ARB6 (GGCCACGCGTCGACTAGTACNNNNNNNNNNACGCC). The first-round PCR product was subjected to a second round of PCR with primers JZ320–2 (GCGACGCCATCTATGTGT) and ARB2 (GGCCACGCGTCGACTAGTAC). The resulting PCR product was sequenced with primer ARB2. Various insertions were apparent in the *flaA* gene. Mutants for which arbitrary PCR failed were tested for insertions in *flaA* by PCR (e.g., using *flaA* primers ACGCGTCGACTGCTCGAAGATGTTTACGTTCAACTATC [upstream] and CCGCTCGAGTCGACCTAACAAAGATAATACAGATTG [downstream] in combination with JZ320–2 [transposon-specific]).

### Motility.

For *Legionella,* bacterial cultures (optical density at 600 nm > 4.0) were diluted 10-fold, placed on a hemocytometer, and observed under high power by a neutral observer blinded with respect to bacterial genotype. Bacteria were considered motile (as opposed to merely subject to Brownian motion) if numerous bacteria could be observed crossing the gridlines on the hemocytometer. For *E. coli,* bacteria were inoculated at a single point in soft (motility) agar (0.35%), incubated at 37 °C, and observed after ~16 h.

### Electron microscopy.

A stationary-phase culture (5 μl) of *Legionella* was adsorbed onto a carbon-coated grid that had been made hydrophilic by a 30-s exposure to a glow discharge in an Edwards Auto 306 vacuum evaporator (http://www.bocedwards.com). Excess liquid was removed with a filter paper (Whatman number 1), and the samples were stained with 1% uranyl acetate for 1 min. The grids were examined using a Tecnai 12 Bio Twin (FEI, Hillsboro, Oregon, United States) transmission electron microscope.

### Western blotting.

Stationary-phase bacteria (25 × 10^6^, or trichloroacetic acid [TCA] precipitates of supernatant from 0.5 ml of stationary-phase culture) were pelleted and resuspended in SDS-containing loading buffer. Proteins were separated on a 10% precast SDS-PAGE gel (Bio Rad, Hercules, California, United States), and transferred to PVDF membrane (Amersham Biosciences, Little Chalfont, United Kingdom). Blots were probed with a primary mouse monoclonal anti-flagellin antibody (hybridoma supernatant, kind gift of M. Swanson, University of Michigan, Ann Arbor, Michigan, United States) and a secondary anti-mouse HRP-conjugated antibody (Amersham Biosciences), and flagellin was visualized using enhanced chemiluminescence (PerkinElmer, Wellesley, California, United States).

### *Legionella* growth curves.

*Legionella* growth in bone marrow–derived macrophages was assayed as described previously [10].

### Cytotoxicity assays.

The Cytotox assay (Promega, Madison, Wisconsin, United States) was used to measure LDH release. In this assay, 1 × 10^5^ macrophages were plated per well of a 96-well plate. Two-fold dilutions of *Legionella* bacteria were grown overnight in liquid buffered-yeast-extract culture and, at the time of infection, samples were matched as closely as possible for optical density (600 nm). In experiments using *Salmonella* or *E. coli,* bacteria were grown to midlog phase in Luria-Bertani media before being used to infect macrophages. Bacteria *(Legionella, Salmonella,* or *E. coli)* were added to wells at the indicated MOI and the plate was then spun at 400 *g* (1,400 rpm) for 10 min, except where indicated. After 30 min of incubation at 37 °C, the media was removed from the infected cells and replaced with fresh media containing 50 μg/ml of gentamicin. After an additional 3.5 h of incubation at 37 °C, LDH release was calculated as a percentage of detergent-lysed macrophages for each strain of mouse. Alternatively, neutral red (Sigma, St. Louis, Missouri, United States) was added to macrophages 2 h after infection at a final concentration of 15 μg/ml. Two hours later, viable macrophages (positive for neutral red) and dead macrophages (unstained) were enumerated by light microscopy. Permeability to ethidium bromide-2 homodimer, indicative of cell death, was assessed using the Live/Dead kit (Invitrogen, Carlsbad, California, United States) according to the manufacturer's instructions.

## Supporting Information

Figure S1 *Salmonella* Induction of Cell Death in Infected Macrophages The indicated strains were used to infect B6 or B6.A-Chr13 macrophages. (Infection and LDH release of macrophages was assayed as shown in Figure 3 and as described in Materials and Methods). The *fljB fliC* mutant strain does not express either *fljB* or *fliC* (the two *Salmonella* flagellin genes). The *invA* mutant is defective in SPI-1 type III secretion. The *flhD* gene is required for expression of the entire flagellar regulon. (A) MOI of 2. (B) MOI of 10.
(52 KB PPT)

### Accession Numbers

The GenBank (http://www.ncbi.nlm.nih.gov/Genbank) GI numbers for the genes and gene products discussed in this paper are *Birc1e* (26245344), caspase-1 (6753282), E. coli MG1655 *fliC* (B1923), *Ipaf* (74180700), *L. pneumophila ahpC* (19880966), *L. pneumophila flaA* (52627363), *L. pneumophila fliI* (1938359), *MyD88* (31543276), *S. flexneri fliC* (30063365), S. typhimurium LT2 *fljB* (1254294), S. typhimurium LT2 *fliC* (1253480), S. typhimurium LT2 *invA* (16421444), and TLR5 (7648686).

## Abbreviations

B6: C57BL/6J
BCYE: buffered charcoal yeast extract
LDH: lactate dehydrogenase
LLO: listeriolysin O
LRR: leucine rich repeats
MOI: multiplicity of infection
NBD: nucleotide-binding domain
NIH: National Institutes of Health
TCA: trichloroacetic acid
TLR5: toll-like receptor 5
